# Spatial Transcriptomic Technologies

**DOI:** 10.3390/cells12162042

**Published:** 2023-08-10

**Authors:** Tsai-Ying Chen, Li You, Jose Angelito U. Hardillo, Miao-Ping Chien

**Affiliations:** 1 Department of Molecular Genetics, Erasmus University Medical Center, 3015 GD Rotterdam, The Netherlands; t.chen@erasmusmc.nl (T.-Y.C.); l.you@erasmusmc.nl (L.Y.); 2 Erasmus MC Cancer Institute, 3015 GD Rotterdam, The Netherlands; j.hardillo@erasmusmc.nl; 3 Oncode Institute, 3521 AL Utrecht, The Netherlands; 4 Department of Otorhinolaryngology, Head and Neck Surgery, Erasmus University Medical Center, 3015 GD Rotterdam, The Netherlands

**Keywords:** spatial omics technologies, NGS-based spatial profiling, probe-based spatial profiling, imaging-based spatial profiling, image-guided spatially resolved single cell sequencing

## Abstract

Spatial transcriptomic technologies enable measurement of expression levels of genes systematically throughout tissue space, deepening our understanding of cellular organizations and interactions within tissues as well as illuminating biological insights in neuroscience, developmental biology and a range of diseases, including cancer. A variety of spatial technologies have been developed and/or commercialized, differing in spatial resolution, sensitivity, multiplexing capability, throughput and coverage. In this paper, we review key enabling spatial transcriptomic technologies and their applications as well as the perspective of the techniques and new emerging technologies that are developed to address current limitations of spatial methodologies. In addition, we describe how spatial transcriptomics data can be integrated with other omics modalities, complementing other methods in deciphering cellar interactions and phenotypes within tissues as well as providing novel insight into tissue organization.

## 1. Introduction

Since the first development of single cell RNA sequencing (scRNAseq) [1], it has been broadly applied to study cellular heterogeneity at the single cell level in tissues. Unlike bulk RNAseq, scRNAseq allows whole transcriptome analysis at single cell resolution, yielding insights into complex cellular heterogeneity in tissues, and, at the same time, revolutionizing the discovery and understanding of cell types as well as their functional cell states and plasticity upon external stimuli [1,2,3,4,5,6,7]. Building on the foundation of scRNAseq, other single cell omics modalities, like single cell genomics, epigenomics and proteomics, have sprawled accordingly [8,9]. Despite the great advances and ongoing success, scRNAseq and other single cell omics modalities cannot be applied in situ, resulting in the loss of information about how cells interact and organize across the tissue landscape. Cellular organization in tissues is closely linked to biological function: in developmental biology, for example, symmetry breaking between daughter cells and cell fate decisions are related to the spatial relationships among cells [10]. Furthermore, in clinical biology, histopathology is used to characterize abnormal spatial organization within tissues, for instance, to assess tumor stages or therapy selection. Although in situ hybridization (ISH) [11] and immunohistochemistry-type methods [12] allow mapping of DNA, RNA or proteins within tissues, only a number of genes or proteins can be analyzed at a time. The modern concept of spatial omics, particularly spatial transcriptomics, was first introduced by Ståhl et al. in 2016, and has now been commercialized by 10x Genomics as the Visium platform. The method relied on capturing polyadenylated mRNAs released from tissue sections onto a barcoded array surface [13]. Since then, the technological development of spatial omics has advanced exponentially, assaying from hundreds to a thousand genes, with even whole genome, transcriptome or proteome profiling becoming possible [8,13,14,15,16]. Moses et al. 2022 provided a thorough review about the historical development of spatial biology technologies [17]. These spatial omics methods are continuously being optimized or emerging to satisfy demands in, among others, the number of detected genes or proteins, sensitivity, resolution, ease of operation and area size. The combination of these new methodologies—that query the whole transcriptome—and traditional methods (like ISH)—that maintain spatial information—expedites new discoveries in various fields, including cancer, immunology, developmental biology and neuroscience. In this review we mainly discuss the existing spatial transcriptomic technologies as well as some other key omics modality techniques. We also introduce the applications of these methods and new emerging technologies in the field. The details of the analysis of spatial omics data are not included in this review as they are described in other reviews [8,16,18].

## 2. Spatial Transcriptomic Technologies

**Figure 1:**
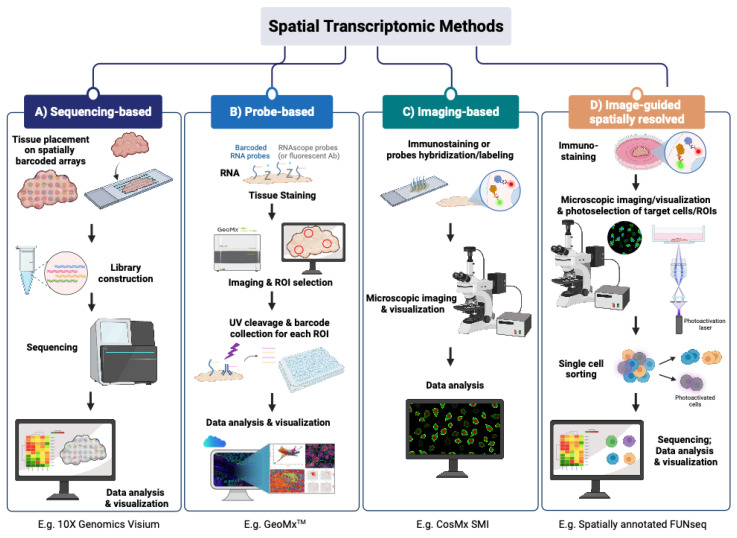
Four main types of spatial transcriptomic methods. Sequencing-based methods (method (**A**)) use barcoded DNA arrays to capture polyadenylated RNA transcripts from tissues followed by next-generation sequencing. Probe-based methods (method (**B**)) capture user-defined targeted transcripts in manually selected regions of interest (ROIs), using corresponding, barcoded oligonucleotide-conjugated probes and can be demultiplexed accordingly afterwards. Imaging-based methods (method (**C**)), similar to probe-based methods, rely on in situ hybridization but with complimentary fluorescent probes, and the targeted transcripts can be detected in a cyclic manner. Image-guided spatially resolved scRNAseq methods (method (**D**)) can select spatially different single cells in ROIs (i.e. photoactivation of single cells in ROIs) followed by fluorescence-activated cell sorting and scRNAseq, thereby preserving their native spatial information as well as retaining in-depth profiling of scRNAseq.

Different spatial transcriptomic methodologies cover various aspects regarding the number of detected genes, resolution and whole transcriptome analysis. These methods are mainly classified as follows: (1) sequencing-based methods; (2) probe-based methods; (3) imaging-based methods; (4) image-guided spatially resolved single cell RNA sequencing methods (Figure 1). The classification was made based on what the final readout of targets is and how spatial contents are obtained: sequencing-based methods rely on whole transcriptome/genome sequencing based on spatially barcoded DNA; probe-based methods require counting of barcoded probes of known targets; imaging-based methods depend on repeated imaging cycles to obtain the final readout of known targets; and image-guided spatially annotated single cell sequencing methods select and isolate single cells in regions of interest (ROIs) based on microscopic images followed by single cell sorting and state-of-the-art single cell omics sequencing.

### 2.1. Sequencing-Based Methods

Sequencing or next-generation sequencing (NGS)-based methods (Figure 1A) are unbiased, as they are similar to single cell whole transcriptome profiling, thus capturing polyadenylated RNA transcripts and thereby enabling the discovery of new differential genes or biological mechanisms. 10X Genomics Visium, Slide-seq, Stereo-seq and Light-seq are examples of such sequencing-based spatial transcriptomic technologies.

*10X Genomics Visium* is adapted from the method reported in Stahl et al. 2016 [13], 10X Genomics Visium. It enables the capture of whole transcriptomes from tissue slices. The method is based on the capture of polyadenylated RNAs on spatially barcoded microarray slides (>1000 spots) followed by reverse transcription, via which the captured transcripts can be mapped back to their original spots. Using this approach, large tissue areas can be investigated in an unbiased manner, without pre-selection of regions or genes of interest. The resolution of Visium is limited to its spot size (55 μm in diameter with 100 μm center-to-center distance), which captures ~a number of cells per spot; therefore, the method does not yield single cell resolution. Both the resolution and the sensitivity of Visium have increased (capture > 10,000 transcripts per spot) when compared to the first reported results [13]. This method can be applied to fixed or frozen tissue slices and has been widely used in various fields, including immunology [19], cancer immunology [20] and neuroscience [21].

*Slide-seq* is similar to Visium, Slide-seq [22], another sequencing-based technology. It uses randomly barcoded microparticles (or “beads”) on a slide to capture RNA transcripts but with improved resolution (10 µm) and sensitivity (~500 transcripts per bead). The method was first applied to whole transcriptome profiling of mouse traumatic injury brain tissue but has since then been applied to other areas, like developmental biology [23], neuroscience [24,25,26] and oncology [27]. Slide-seq has been shown to identify similar subpopulations of cells as those found in scRNAseq [22]. Moreover, the improved version of Slide-seq, Slide-seqV2, has demonstrated even higher spatial resolution (near cellular resolution) and sensitivity (>1000 transcripts per bead) and could identify underlying genetic programs that were poorly sampled with Slide-seq [28].

Both Visium and Slide-seq methods have been shown to improve cell (sub)typing by integrating the spatial transcriptome data with scRNAseq data [29]. Various integration and cell-type decomposition methods were introduced to accommodate this, including probability-based methods [29,30], graph-based methods [31] and deep learning-based methods [32,33].

*Spatio-temporal enhanced resolution omics sequencing (Stereo-seq)*, a newly developed NGS-based spatial profiling method, uses randomly barcoded DNA nanoballs deposited in arrays to achieve nanoscale resolution, thereby reaching ~single cell resolution. The method enables large field-of-view (FOV) (cm × cm) spatial transcriptomics at ~cellular resolution and has been applied to mouse organogenesis [34], plant biology [35], neuroscience [36] and developmental biology [37].

*Light-seq*, another newly developed NGS-based spatial omics method, uses light-directed DNA barcoding in fixed cells and tissues followed by ex situ NGS sequencing [38]. The method enables transcriptomic profiling of large or small ROIs in intact fixed tissue samples based on location, morphology or protein stains, without cellular dissociation. Although the method is not yet at single cell resolution, it has been applied to discover biomarkers of dopaminergic amacrine cells, a very rare cellular subtype, in mouse retinal sections.

Despite the advances in NGS-based methods, most of them lack single cell resolution or have relatively low transcript counts, reducing their sensitivity to low abundance transcripts [13,15,39,40]. These methods often capture transcripts using a fixed array of barcoded primers and uniformly sample transcripts across the entire sample. In contrast, defining ROIs based on microscopically observable features could be highly advantageous, as this allows researchers to specifically sample and study these ROIs in higher detail and with higher (single cell) resolution as well as to have higher transcript counts, like image-guided spatially resolved single cell sequencing [41,42,43,44] (see “Image-guided spatially resolved single cell sequencing” section for detail).

### 2.2. Probe-Based Methods

In contrast to NGS-based spatial transcriptomic methods, oligonucleotide probe-based methods like NanoString’s digital spatial profiling system were designed to capture targeted transcripts in manually selected ROIs [45,46]. Targeted transcripts can be uniquely bound to their corresponding, barcoded oligonucleotide-conjugated probes and can be demultiplexed accordingly afterwards.

*NanoString’s digital spatial profiling system, GeoMx^TM^*, uses in situ hybridization probes, consisting of target complimentary oligonucleotide sequences linked to indexing oligonucleotide barcodes via a UV-photocleavable linker (Figure 1B). Upon UV light illumination, the barcodes can be photocleaved and collected via the GeoMx Digital Spatial Profiler (DSP) instrument. This method enables profiling of RNA targets (or proteins) in ROIs. The system offers RNA probe sets (ACD’s RNAscope probes) targeting 18,000+ transcripts of human protein coding genes or targeting 21,000+ transcripts of mouse protein coding genes. Thin tissue slices (~5–10 μm), like formalin-fixed, paraffin-embedded (FFPE) tissue sections or fresh frozen tissues, will be first incubated with the probes overnight followed by stringent washes and the addition of fluorescently labelled antibodies (as morphology markers). User-defined ROIs (~20–300 cells/ROI) are then profiled on the GeoMx DSP via photo-cleaving and collecting photocleavable barcoded probes. Cleaved indexing probes are then quantified using NanoString nCounter Technology, via which digital quantification of RNA expression with spatial context will be generated [46]. Despite the great advantages of high-sensitivity and spatially resolved technology, nonetheless it is of low throughput and not at single cell resolution.

### 2.3. Imaging-Based Methods

Similar to the probe-based spatial transcriptomic method, imaging-based methods also rely on in situ hybridization, but with complimentary fluorescent probes (Figure 1C). Initially limited in the number of detected transcripts, breakthroughs have been made in recent years, enabling multiplexing with sequential rounds of hybridization and imaging. Here we discuss the commonly used imaging-based methods.

*NanoString’s newer system, CosMx™ spatial molecular imager (CosMx SMI)* enables quantification and visualization of up to 1000 targeted transcripts and 64 protein analytes via automated, cyclic fluorescence, in situ hybridization [47]. CosMx can profile up to 1 million cells per sample (4 samples per run) and spatially profile targeted molecules at 3D subcellular resolution. This method requires a pre-selected probe panel and, therefore, is not whole transcriptomic profiling.

*Multiplexed error-robust fluorescence* in situ *hybridization (MERFISH)* combines fluorescence in situ hybridization with combinatorial labels to enable sequential rounds of hybridization and imaging. The serial images are then decoded using error-robust barcodes that are designed to label specific RNA molecules [48,49,50]. This method allows detection ~10,000 genes at sub-cellular resolution.

Similar to MERFISH, *sequential fluorescence in situ hybridization (seqFISH and seqFISH+)* is able to detect ~10,000 genes at sub-cellular resolution by combining colors into pseudocolors [14,51,52], and, therefore, can be applied to investigate intracellular organization [53].

*Spatially-resolved transcript amplicon readout mapping (STARmap)* [54] integrates hydrogel–tissue chemistry, targeted signal amplification (DNA amplicons) and in situ sequencing to identify and quantify spatially distributed RNAs via 3D imaging. STARmap can detect 160 to 1020 genes simultaneously in sections of mouse brain at single cell resolution.

Most imagining-based methods are featured on single cell resolution, high efficiency, specificity and experimental reproducibility. Even though, pre-selected targeted transcripts need to be determined in advance and the FOV of tissues is limited [54].

### 2.4. Image-Guided Spatially Resolved Single Cell Transcriptomic Sequencing

Despite the great advances in spatial omics, most of the existing methods lack in-depth single cell whole transcriptomic profiling, analyzing tens of thousands of genes per cell. Image-guided or microscopy-based methods have been combined with state-of-the-art scRNAseq to address this limitation. These image-guided spatially resolved scRNAseq methods (Figure 1D) can select spatially different cells or regions of interest followed by scRNAseq, thereby preserving their native spatial information as well as retaining in-depth profiling of scRNAseq. The earliest example is laser capture microdissection (LCM), which physically microdissects ROIs of tissue sections with UV or infrared light [55,56]. *Geographical position sequencing (Geo-seq)* has combined LCM and scRNAseq to investigate the spatial transcriptome of mouse early embryo, mouse brain and pathological liver and sperm tissues [44]. *Zipseq* [57], another example, uses photocaged oligonucleotide to print barcodes onto live cells in intact tissues and was able to identify new gene expression patterns associated with histological structures like tumor microenvironment.

**Figure 2:**
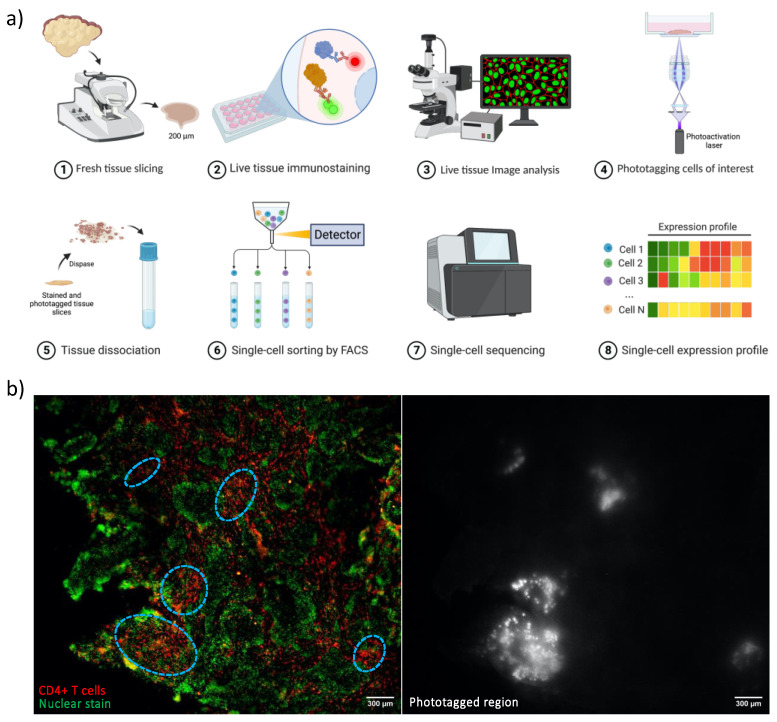
(**a**) The pipeline of spatially annotated FUNseq. (**b**) Live HNSCC tumor tissue slice imaging on the UFO microscope. Left panel: SPY505 nuclear staining (green) and CD4 immunostaining (red) of the tissue were imaged; right panel: Phototagged image, where the regions with a higher density of CD4+ T cells (highlighted in blue circles in the left panel) were phototagged (higher intensity). Scale bar = 300 μm.

*NICHE-seq* [43] and *spatially annotated functional single cell sequencing (FUNseq)* [41,42], on the other hand, use photoactivatable fluorescent reporters/dyes and light microscopy to photolabel spatially different cells followed by fluorescence-activated cell sorting and scRNAseq, thereby linking spatial information with the transcriptomically profiled single cells. *NICHE-seq* has been applied to identify rare niche-specific immune subpopulations and gene programs in infected B cell follicles and tumors [43]. *Spatially annotated FUNseq*, developed in our group, has been applied to unravel intratumoral heterogeneity and decipher the tumor invasiveness of cells located in the outermost edge of a cell mass [41]. In addition, our group has recently applied spatially annotated FUNseq to live clinical tumor tissue sections (Figure 2a), following these steps: (1) tumor tissues are sliced into ~200 μm sections; (2) live tissue slices are immunostained against proteins of interest (i.e., CD4) and/or stained with nuclear dyes as well as photolabeling dyes used in FUNseq [41,42]; (3) the (immuno)stained tissue slices are then visualized and imaged via fluorescence microscopy to determine cells of interest or regions of interest in tissue slices; (4) cells of interest or regions of interest, defined in Step 3, are photolabeled (or phototagged) [41,42]; (5) tissue slices are then dissociated into a single cell suspension followed by (6) fluorescence-activated cell sorting (FACS); (7) the sorted cells can be subjected to single cell (DNA/RNA) sequencing or single cell proteomic profiling [58] followed by (8) single cell analysis.

Image-guided spatially resolved single cell transcriptomic sequencing enables in-depth single cell whole transcriptomic profiling by integrating state-of-the-art scRNAseq with live cell imaging. Our spatially annotated FUNseq, in particular, allows imaging a large FOV (up to 1.28 cm × 1.28 cm) of live clinical tissue sections while maintaining single cell/subcellular resolution using our ultrawide field-of-view optical (UFO) microscope [41,42]; it also enables phototagging single cells of interest in the imaged FOV simultaneously, without the cell number limitation of photolabeling. In addition, this method can be potentially used to monitor and profile live cells displaying cellular dynamics of interest. Figure 2b shows a representative live tissue image of human head and neck squamous cell carcinoma (HNSCC) acquired on our UFO microscope, where the tissue slice was stained with a SPY505 nuclear dye and immunostained against CD4 proteins (targeting CD4+ T cells). The regions with a higher density of CD4+ T cells were phototagged [41,42]. Spatially annotated FUNseq can be combined with any existing single cell omics of choice as FUNseq itself has been successfully integrated with single cell whole transcriptome [41,42], single cell whole proteome [58] and single cell whole genome profiling (unpublished data).

These photoactivation-based spatial profiling methods are reproducible, sensitive and specific, but there is room for improvement regarding throughput (i.e., more photoactivation panels or multiplexed labeling).

## 3. Different Spatial (Multi-) Omics Modality Techniques

### 3.1. Spatial Genomics and Transcriptomics

With the advent of improved resolution and sensitivity of spatial transcriptomic technologies, integration with other omics modalities can provide broader and more comprehensive information on tissue characterization. The combination of spatial genome sequencing with in situ transcriptomic profiling could shed light onto our understanding of how genome organization and function are programmed. With the advent of spatial genomic sequencing in intact tissues [59], integration of the gene expression profile with genomic data has been made feasible. *Slide-DNA-seq* [60], a method for capturing spatially resolved DNA sequences from tissue sections, enables the discovery of distinct tumor clones and their copy number alterations. This method has been integrated with spatial transcriptomics to unravel genes associated with clone-specific genetic aberrations and/or the local tumor microenvironment [60].

### 3.2. Spatial Proteomics and Transcriptomics

Spatial proteomics enable profiling protein expression within individual cells at subcellular resolution, resolving spatiotemporal protein locations as well as protein–protein interactions [61]. Most of the probe-based and imaging-based spatial transcriptomics methods also enable spatial proteomic profiling through oligonucleotide- or fluorescence-conjugated antibodies against proteins of interest. By integrating proteomic data with spatial transcriptome analysis, biological processes that are not captured by spatial transcriptomics, like post-translational modifications, could be unraveled. *Deterministic barcoding in tissue for spatial omics sequencing (DBiT-seq)* [62], an NGS-based spatial transcriptomic technology, also allows profiling proteins of interest in formaldehyde-fixed tissues. The method delivers DNA barcodes to tissue sections via parallel microfluidic channels followed by in situ reverse transcription and library preparation. Proteins can be co-measured through the same platform by applying antibody-derived DNA tags to the fixed tissue slide prior to flow barcoding, similar to Ab-seq [63] or CITE-seq [64]. Using the multi-omic modalities of DBiTseq, major tissue types in early organogenesis of mouse embryos as well as fine features like microvasculature in a brain and pigmented epithelium have been revealed [62]. In addition, two spatial transcriptomics and proteomics integrated methods, (Spatial PrOtein and Transcriptome Sequencing (SPOTS) [65] and spatial multi-Omics (SM-Omics) [66]), showed that Visium enabled whole transcriptome/proteome co-profiling using fluorescence-conjugated antibodies, through which 21 proteins in mouse spleen and breast cancer samples as well as 6 proteins in mouse brain, spleen and colorectal cancer samples were visualized.

### 3.3. Mass Spectrometry-Based Methods for Spatial Proteomics, Lipidomics and Metabolomics

Mass spectrometry-based methods, like *mass spectrometry imaging (MSI)* [67,68], *multiplexed ion beam imaging (MIBI)* [69,70] *and matrix-assisted laser desorption/ionization (MALDI) imaging mass spectrometry (IMS)* [71,72,73], are techniques that combine the ability of microscopy to provide spatial information with the specificity of mass spectrometry (MS) for unlabeled mapping of proteins and metabolites in biological tissues.

Significant improvements have been made in MSI and spatially resolved MS-analysis as a whole over the past decade, and many of these have focused on enabling spatial proteomics and metabolomics at a single cell level [68]. MIBI, for example, provides high-resolution spatial maps of protein expression in tissues by combining mass spectrometry with immunofluorescence staining imaging [69,70]. MIBI involves the use of metal isotope-labeled antibodies to target specific proteins, which are then detected and quantified using a mass spectrometer. MIBI enables analysis of up to 100 markers with unique signals of each antibody without spectral overlap. Furthermore, MALDI-IMS has been demonstrated to simultaneously determine the distribution of hundreds of molecules (i.e., proteins, lipids and metabolites) from tissue sections with high spatial resolution [71,72,73]. For example, in Good et al. 2022, MALDI-IMS was applied to spatially profile lipid distributions in fresh frozen bone tissues at 10 μm resolution [71]. And in Andersen et al. 2021, this method has been applied to spatially detect metabolites and lipids on prostate tissues [73].

### 3.4. Non-Mass Spectrometry-Based Methods for Spatial Proteomics

In addition to mass spectrometry-based methods, other high-throughput spatial proteomic profiling methods, such as *co-detection by indexing (CODEX)* [74] and *tissue cyclic immunofluorescence (t-cyCIF)* [75], provide supreme resolution of the proteome within tissue sections. Both methods are imaging-based technologies, which allow cyclic detection of tens of proteins via microscopic fluorescence imaging, either by DNA-indexed antibodies followed by complimentary DNA-conjugated fluorescent dyes (CODEX) or by fluorescent dye-conjugated antibodies (t-cyCIF). These methods have been applied to various samples and disease models, including colorectal cancer [76], mouse spleen [74] and tumor immunology [75].

### 3.5. Spatial Epigenomics

Spatial epigenomics captures epigenomic modifications, including DNA methylation, histone modifications and chromatin accessibility, in tissue sections. Spatially resolved chromatin accessibility profiling of tissue sections using next-generation sequencing (*spatial-ATAC-seq*) [77] and combining in situ Tn5 transposition chemistry and microfluidic deterministic barcoding, has been applied to profile mouse embryos and delineate tissue region-specific epigenetic landscapes as well as identify gene regulators involved in the development of the central nervous system [77]. *Spatial-CUT&Tag* (cleavage under targets and tagmentation) enables spatially resolving of genome-wide profiling of histone modifications by combining in situ CUT&Tag chemistry, microfluidic deterministic barcoding and next-generation sequencing [78]. *Epigenomic MERFISH* enables spatially resolved single cell epigenomic profiling and has been demonstrated to map active promoters and putative enhancers in the mouse brain [79].

Technological advances that enable the integration of different spatial modalities will greatly improve our ability to study tissue complexity and our understanding of biological processes in greater detail.

Features of the spatial omics methods mentioned in this review are summarized in Table 1.

## 4. Novel Biological Insights Resolved by Spatial Omics Technologies

Spatial omics can be utilized to deepen our understanding of tissue complexity as the technology provides thorough information of cell (sub)types, cell–cell interactions and heterogeneity of individual cells in intact tissues.

The methods have been widely applied in the fields of oncology, cancer immunology and developmental biology. For example, diverse heterogeneity of tumor-associated macrophages (TAMs) and their role in influencing the tumor microenvironmental landscape was better understood via spatial omics profiling [80,81]. The metastatic liver cancer microenvironment was found to undergo spatial reprogramming of immunosuppressive cells like a subtype of macrophage MRC1+ CCL18+ M2-like macrophages, which harbored enhanced metabolic activity and contributed to cancer metastasis [81].

In developmental biology, Spatial-CUT&Tag has been applied to reveal epigenetic control of cortical layer development and spatial patterning of cell types determined by histone modification in the mouse brain, thereby dissecting the underlying mechanisms of how spatial epigenetic patterns modulate gene expression and tissue development [78].

In cardiac research, spatial omics technologies have been applied to the investigation of heart development and injury [82]. For instance, the combination of MALDI-IMS and label-free proteomics on the same section of tissues was applied to identify protein profiles of cardiac ischemia/reperfusion myocardial damage in rats [83]. In this way, alterations in cytoskeleton reorganization and inflammation within the infarct tissue were found [83]. Furthermore, by combining spatial transcriptomics and single cell transcriptomics, spatial structural alternations and cell–cell interactions during chicken heart maturation were also revealed [84].

## 5. Conclusions, Challenges and Future Perspectives

In order to comprehend the intricate molecular mechanisms of cellular interactions within tissues, emerging spatial omics technologies compete in terms of owing higher resolution (single cell or subcellular resolution), faster throughput, better sensitivity, deeper coverage, greater multiplexity, more user-friendly procedures and greater versatility of assayed samples (i.e., FFPE, fresh frozen and live tissues). Owing to the rapid development of these various aspects in the spatial transcriptomics field, a deeper understanding of cellular states and functions within tissues has been greatly noted. The newer invention of spatial genomics, epigenomics, metabolomics and proteomics further complements the findings made by spatial transcriptomics.

With future development in the spatial omics field, systematically assaying larger tissue areas for 3D reconstruction as well as deciphering transcriptomic (or other omic) changes over time may be possible. To reach this, NGS-based methods need improved spatial resolution and probe-based and imaging-based methods require an increase in profiling speed and the number of targets that can be profiled. Image-guided spatially resolved methods need to improve either the profiling speed (i.e., Geo-seq) or the multiplexing capacity (i.e., NICHE-seq and spatially annotated FUNseq). In addition, transcriptomic (or other omic) variations associated with (time-dependent) changes in cellular dynamics or interactions in response to treatments or the surrounding microenvironment may be feasible when technologies for spatial profiling of live tissues (i.e., NICHE-seq and spatially annotated FUNseq) are made more mature. Furthermore, developments in both 3D reconstruction and temporal profiling require more advanced microscopic imaging systems tailored to these applications as well as associated visualization analysis frameworks.

In addition to addressing the technological challenges, development of new computational tools and algorithms will be accompanied. Current challenges of spatial omics data analysis include, amongst others, how to properly normalize data matrices and remove low quality data, how to increase the signal-to-noise ratio, how to or whether to smooth data to improve sensitivity and how to remove unwanted technical and biological variations. Furthermore, more and comprehensive integration strategies across different modalities are expected to be developed, for instance, integrating spatial metabolomics with other modalities. The development of algorithms or analysis packages that are robust for each technology as well as compatible with other platforms is crucial to increase the applications of these technologies.

Another challenge for the spatial omics field will be to create novel, yet validated, models about how multi-cellular spatial networks communicate and orchestrate biological functions. For example, will traditionally identified cell types be seen as new cell types in different multi-cellular structures or patterns at the tissue level? How do microenvironmental cues reprogram cell or tissue fate? Leveraging on the upcoming big spatial omics data and existing single cell omics profiles, new realization of how cells communicate between each other at the tissue level, how spatial organization and patterns influence biological functions and how environmental cues impact (multi-) cellular development will be made.

These deeper biological insights will enable a comprehensive understanding of spatial patterns, cellular interactions and tissue architectures as well as unraveling novel mechanisms of disease formation and organism development.

## Figures and Tables

**Table 1:** Comparison table of the selected spatial omics technologies.

Categories of Methods	Name	Single Cell Resolution	Omics Type *	Whole Transcriptome Profiling	Tissue Type *
**Sequencing-based**	10X Genomics Visium	~55 µm/spot	RNA	x	FFPE, FF
	Slide-Seq	~10 µm/spot	RNA	x	FFPE, FF
	Stereo-seq	Close to single cell	RNA	x	FFPE, FF
	Light-seq	Close to single cell	RNA	x	FFPE
**Probe-based**	NanoString GeoMx	~20–300 cells/ROI	RNA, protein	~hundreds of targets	FFPE, FF
**Imaging-based**	NanoString CosMx	x	RNA, protein	~1000 targets	FFPE, FF
	MERFISH	x	RNA, protein	~10,000 targets	FFPE, FF
	seqFISH	x	RNA, protein	~10,000 targets	FFPE, FF
	STARmap	x	RNA	~100–1000 targets	FFPE, FF
**Image-guided spatially resolved single cell transcriptomic sequencing**	Geo-seq	a number of cells	RNA	x	FFPE, FF
	Zipseq	x	RNA	x	Live tissue, (FF)
	NICHE-seq	x	RNA	x	Live tissue, (FF)
	Spatially annotated FUNseq	x	RNA, (DNA, protein)	x	Live tissue, (FF)
**Different modalities** **and others**	Slide-DNA-seq	~10 µm/spot	DNA	Single cell whole genome sequencing	FFPE, FF
	DBiT-seq	~10 μm/pixel	RNA, protein	x	FFPE, (FF)
	MIBI	x	Protein, metabolite	~100 targets	FFPE
	MALDI-IMS	~10 μm/pixel	Protein, lipid metabolite	>100 targets	FF, (FFPE)
	CODEX	x	Protein	~60 targets	FFPE, FF
	t-cyCIF	x	Protein	~60 targets	FFPE
	spatial-ATAC-seq	~20 μm/pixel	Chromatin accessibility	Genome-wide chromatin accessibility	FF, (FFPE)
	Spatial-CUT&Tag	~20 μm/pixel	Histone modification, (RNA, protein)	Genome-wide profiling of histone modifications	FF, (FFPE)
	Epigenomic MERFISH	x	Histone modification	Genome-wide profiling of histone modifications	FFPE, (FF)

* Tissue type: FFPE, formalin-fixed paraffin-embedded; FF, fresh frozen. * Tissue or omics type in brackets indicates application has not been shown directly in peer-reviewed publications but should be possible based on theoretical considerations or pre-publication data.

## Data Availability

Data is unavailable due to ethical restrictions.
